# Immunity and the carotid body: implications for metabolic diseases

**DOI:** 10.1186/s42234-020-00061-5

**Published:** 2020-12-23

**Authors:** Silvia V. Conde, Joana F. Sacramento, Fatima O. Martins

**Affiliations:** iNOVA4Health, CEDOC, NOVA Medical School, NMS, Universidade Nova de Lisboa, Rua Câmara Pestana, n°6, Edifício 2, piso 3, 1150-274 Lisbon, Portugal

**Keywords:** Carotid body, Carotid sinus nerve, Vagus nerve, Parasympathetic nervous system, Sympathetic nervous system, Immunity, Inflammation, Metabolic disease

## Abstract

Neuro-immune communication has gained enormous interest in recent years due to increasing knowledge of the way in which the brain coordinates functional alterations in inflammatory and autoimmune responses, and the mechanisms of neuron-immune cell interactions in the context of metabolic diseases such as obesity and type 2 diabetes. In this review, we will explain how this relationship between the nervous and immune system impacts the pro- and anti-inflammatory pathways with specific reference to the hypothalamus-pituitary-adrenal gland axis and the vagal reflex and will explore the possible involvement of the carotid body (CB) in the neural control of inflammation. We will also highlight the mechanisms of vagal anti-inflammatory reflex control of immunity and metabolism, and the consequences of functional disarrangement of this reflex in settlement and development of metabolic diseases, with special attention to obesity and type 2 diabetes. Additionally, the role of CB in the interplay between metabolism and immune responses will be discussed, with specific reference to the different stimuli that promote CB activation and the balance between sympathetic and parasympathetic in this context. In doing so, we clarify the multivarious neuronal reflexes that coordinate tissue-specific responses (gut, pancreas, adipose tissue and liver) critical to metabolic control, and metabolic disease settlement and development. In the final section, we will summarize how electrical modulation of the carotid sinus nerve may be utilized to adjust these reflex responses and thus control inflammation and metabolic diseases, envisioning new therapeutics horizons.

## Background

“Neuroinflammation” is a term that generally describes the innate immune inflammatory response generated in the central nervous system (CNS) after injury (Kennedy and Silver 2016). However, in addition some evidences suggest that this brain-immune axis integrates inputs from peripheral neuronal networks to inform immune cells on whole body immune status (Jakob et al. 2020).

In the brain, astrocytes and microglia contribute to homeostasis by supporting not only neuronal function, but also immunological responses such as pathogen destruction and the removal of dead neurons. In fact, astrocytes and microglia are the most abundant producers of cytokines in the brain (Kennedy and Silver 2016). It was presumed that these central inflammatory responses occurred in isolation due to their separation from the periphery by the blood-brain-barrier (BBB) (Coceani et al. 1988). However, it is now generally accepted that CNS communicates with the peripheral immune system through both neural and humoral mechanisms (Kennedy and Silver 2016). Moreover, the discovery of the circumventricular organs (CVOs) revealed further immune-to-brain communication pathways (Blatteis et al. 1987; Stitt 1990). These CVOs have a leaky BBB, and are situated near the CNS areas that react to peripheral immune challenges, such as the area postrema (AP) and the organum vasculosum, which project into the nucleus tractus solitarii (NTS) (Cai et al. 1996). NTS contributes to immunomodulatory functions that regulate fever, breathing and autonomic efferent activity to the cardiovascular system, and other peripheral systems (Zoccal et al. 2014).

Impairment of the immune responses by the brain has also been identified as a contributing factor in the disarrangement of inflammatory processes and thus disease development. In fact, exacerbated inflammation in the brain is involved in type 2 diabetes, obesity, neurodegenerative diseases and many others highly incident diseases (Ferreira et al. 2014; Jais and Brüning 2017; Terrando and Pavlov 2018). Adding to this, the neuro-immune link in the periphery and its dysregulation in inflammatory states such as chronic inflammatory illness, ageing and metabolic diseases, and the process of integration of related peripheral inputs in the CNS are emerging as potential mechanisms of disease and thus targets for therapy. For example, metabolic diseases are associated with chronic “inflammation”, which is characterized by abnormal cytokine production, increased acute-phase reactants and other mediators, and activation of a network of inflammatory signaling pathways. Allied to this, metabolic thermogenesis, blood pressure regulation and intestinal motility are modulated through interactions between sensory nerves, autonomic nervous system, central neural regulators and immune cells (Ordovas-Montanes et al. 2015). In this context, catecholamines derived from macrophages and norepinephrine (NE) released from efferent sympathetic nerves promote thermoregulation by brown adipose tissue (BAT), by activating on adrenergic receptors (ARs) of brown adipocytes, that in turn increase expression of uncoupling protein-1 (UCP1) (Nguyen et al. 2011).

In the present review we will focus on the circuits and mechanisms involved by which the brain and peripheral nervous system communicate with the immune system, giving special attention to this relationship in the context of metabolic disease settlement and development.

## The nervous and immune systems

The nervous system is composed of the CNS (the brain and the spinal cord) and the peripheral nervous system. The peripheral nervous system has somatic and autonomic components. Somatic nerves originate in the CNS, innervate skeletal muscles, and provide voluntary control of movements. The autonomic nervous system has 3 branches: the sympathetic branch originated in the thoracic and lumbar areas of the spinal cord, the parasympathetic branch originated in the cranial nerves and sacral spinal cord, and the enteric system originated in two types of ganglia located in the gastrointestinal tract (Pavlov et al. 2018).

The sympathetic neurons synapse with relatively long postganglionic fibers that innervate blood vessels, lymphoid tissue and organs, bone marrow, joints, spleen, lungs and airways, gastrointestinal tract, liver, kidneys, and other visceral organs (Elenkov et al. 2000; Jänig 2014).

The parasympathetic branch of the autonomic nervous system, the main nerve of which is the vagus, innervates peripheral viscera. The vagus nerve, whose cell bodies reside in the dorsal motor nucleus of the vagus (DMN) and the nucleus ambiguous in the brainstem medulla oblongata, innervate the visceral organs, including the lungs, heart, liver, gastrointestinal tract, kidneys and pancreas, and form synaptic contacts with postganglionic neurons in proximal to or within these organs (Jänig et al. 2017).

The enteric nervous system is a quasi autonomous part of the nervous system, with neural circuits localized in the gut that coordinate gastrointestinal functions (Goyal and Hirano 1996).

**Fig. 1:**
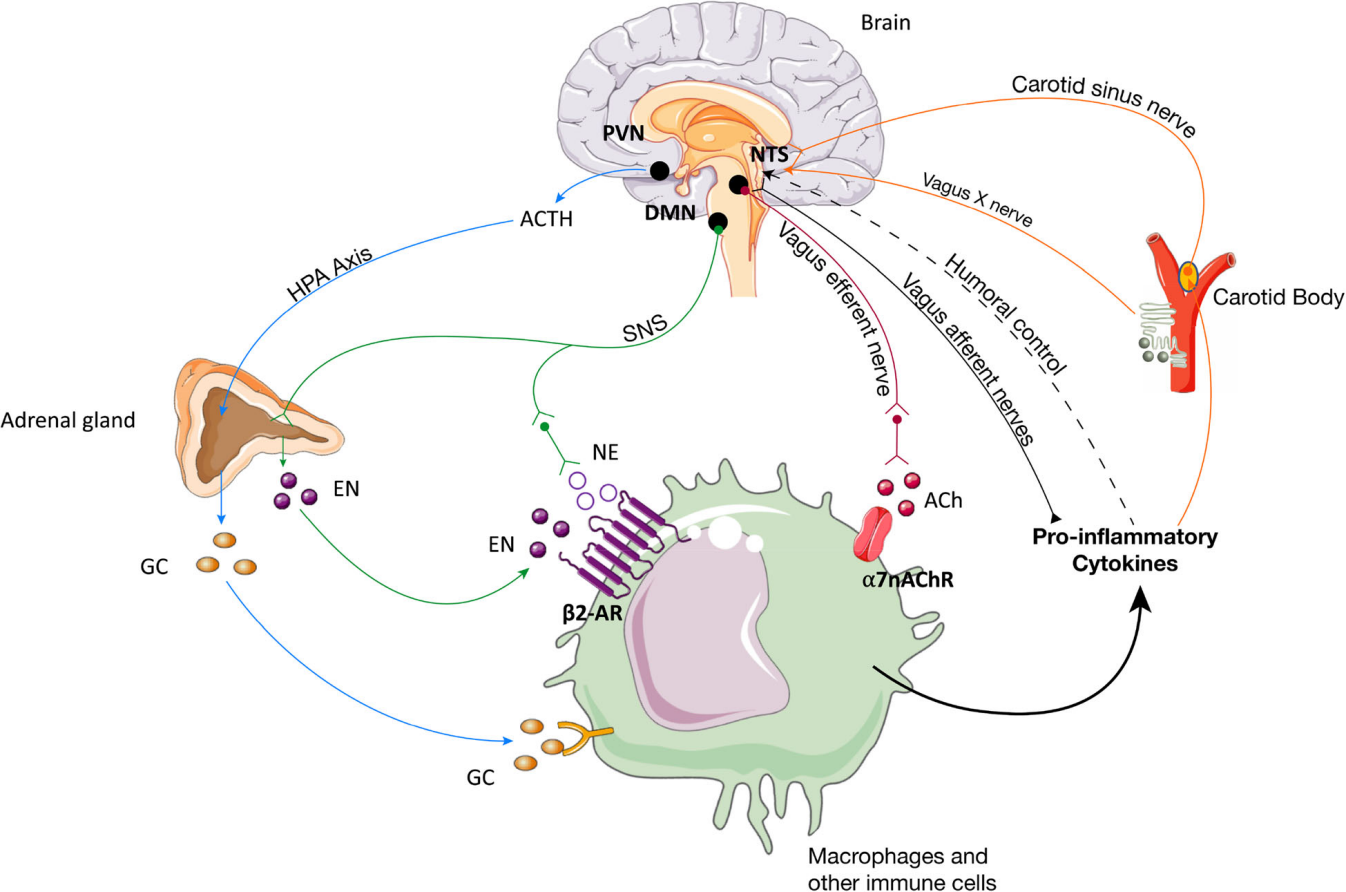
Neuronal reflexes controlling inflammation. The HPA axis is activated by several stimuli to activate the paraventricular nucleus (PVN) of the hypothalamus leading to the release of cortisol releasing hormone (CRH) into the anterior pituitary. In turn, CRH induces the release of adrenocorticotrophic hormone (ACTH) in blood, which stimulate the production of glucocorticoids (GC) by the cortex of the adrenal glands, which are potent anti-inflammatory molecules whose effect is mediated by the signaling via glucocorticoid receptor (GR), a nuclear receptor expressed by almost all cells in the body and in particular in innate immune cells; Vagal anti-inflammatory reflex is characterized by peripheral vagal afferent nerves detecting inflammation and sending this information to the CNS. Reciprocal connections between the NTS and DMN mediate communication with and activation of efferent vagus nerve fibers inducing the release of ACh and ultimately attenuating cytokine production and inflammation through a mechanism, based on the binding of ACh on nicotinic α7nAChR; Additionally, these cytokines can also activate directly this region in the brain by a humoral control in order to revert inflammation; Sympathetic fibers originating in the spinal cord terminate innervate directly visceral organs and immune cells and release norepinephrine (NE) that binds to its specific receptors on these cells. Efferent sympathetic output to the adrenal gland induces the secretion of epinephrine (EP) from chromaffin cells that circulates in blood to reach specific receptors in immune cells. EN and NE joint action stops inflammation and release of inflammatory cytokines; finally, inflammation resolution also occurs through carotid body (CB) action. Briefly, released inflammatory mediators by immune cells activate the CB, which in turn activates carotid sinus nerve (CSN) that projects within the nucleus tractus solitarii (NTS)

The communication between central and peripheral nervous systems occurs in both ways, with efferents nerves bringing information to the periphery and afferent nerves transmitting information from the periphery to the CNS. The vagus nerves are one of the most important afferent nerves and innervate the lungs, heart, gastrointestinal tract, liver, and pancreas and project centrally to the NTS in the brainstem medulla oblongata of the CNS (Fernandez et al. 2014; Mazzone and Undem 2016) (Fig. 1).

In this review we will also discuss the immune system, which is composed by the innate and the adaptive divisions. Innate immune system constitutes the first defense barrier against foreign organisms. In contrast, the adaptive immune system shows a delayed response but at same time presents highly efficient mechanisms to eliminate pathogens and maintain tolerance to a next contact to those pathogens (Pacheco et al. 2012).

T-cells are the central players in the adaptive immune response and are the main promoters of cytokine production. These cytokines will then promote or potentiate functions of other immune cells such as macrophages, dendritic cells (DCs), and natural killer (NK) cells (Pacheco et al. 2012). There is a growing evidence that some cells from the adaptive immune system, specially the T-cells, constitute an important link between the nervous system and the immune system (Mignini et al. 2003). This aids in the regulation of nervous system functions such as acquisition of memory, behavior, neurogenesis, and may contribute to many neurodegenerative disorders (Yirmiya and Goshen 2011). This link is reciprocal, as data suggest that T-cells and the wider immune system can be regulated by neurotransmitters (Franco et al. 2007).

Several receptors for neurotransmitters classically expressed in both the central and peripheral nervous system are also expressed on the surface of immune system cells. For instance, T-cells and DCs express some subtypes of glutamate receptors, acetylcholine receptors (AChRs), serotonin receptors, dopamine receptors, ARs, and others (Pacheco et al. 2010). Many other studies show that neurotransmitter signals modulate the immune response (Glaser and Kiecolt-Glaser 2005). For example, Tracey et al. have demonstrated that activation of α7 nicotinic ACh receptor (α7nAChR) on the macrophages inhibits pro-inflammatory cytokines release (Fig. 1) (Andersson and Tracey 2012), which they named the cholinergic anti-inflammatory pathway (Bonaz et al. 2016; Borovikova et al. 2000). Also, β2-ARs have been shown to be significantly involved in the modulation of the immune system, as administration of exogenous β2-AR agonist, salbutamol, impairs CD8+ priming by cross-presenting DC in mice (Hervé et al. 2013).

Additionally, both anti-inflammatory and pro-inflammatory pathways can be modified in experimental animals by adjusting animal behavior patterns and thus neurotransmitter levels (Cohen et al. 1994).

Cytokines, the main soluble molecules mediating regulatory communications between immune cells, use receptors for cell-to-cell communication that in the past were solely assigned to immune regulation, including pattern recognition receptors (such as toll-like receptors (TLRs)) and receptors for tumor necrosis factor (TNF), interleukin-1beta (IL-1β), and other cytokines. However, these receptors have now been shown to be present on sensory neurons (de Lartigue et al. 2011; Steinberg et al. 2016). Quan et al. also showed that the direct administration of many cytokines in the brain promotes regulation of whole-body inflammation (Quan 2014). For example, IL-1, interferon-gamma (IFN-γ) or TNF-α injection in the brain induced leukocyte infiltration (Ching et al. 2005) while peripheral lipopolysaccharide (LPS) injection induced the expression of IL-1 in the CNS without affecting the entry of leukocytes in the brain (Ching et al. 2006). These and many other studies led to the proposal that peripheral sensory nerves report on inflammation status to the brain and vice-versa (Fernández et al. 2011; Watkins et al. 1995).

Immune cell activation and consequent inflammation is a protective mechanism. However, pro-inflammatory cytokines may also cause tissue injury and have deleterious effects. This occurs, for example, in cases of Immune-Mediated Inflammatory Diseases (IMIDs) such as rheumatoid arthritis (RA), inflammatory bowel disease (IBD) and Systemic Lupus Erythematosus (SLE) (Bradley 2008). Also, many neurodegenerative disorders are associated with dysregulated inflammatory processes. Numerous studies have shown that Alzheimer’s and Parkinson’s disease are accompanied by excessive levels of pro-inflammatory cytokines in both the peripheral nervous system and brain (Guzman-Martinez et al. 2019). Additionally, persistent or exaggerated inflammatory pathway activation has been connected to the most significant metabolic diseases such as type 2 diabetes, obesity, insulin resistance and other metabolic pathologies, which we will focus on here. One of the organs that have been considered to underlie the inflammatory response associated with metabolic and associated cardiovascular diseases is the white adipose tissue (WAT), and in particular the visceral WAT (Hajer et al. 2008). The visceral WAT secretes pro-inflammatory cytokines, such as Interleukin 6 (IL-6), IL-8, MCP-1, PAI-1, as well as complement factors and substances involved in systemic acute phase response and innate immunity (Lee and Fried 2010). During obesity there is an increase in the number of macrophages within the tissue, which are the source of TNF-α and other pro-inflammatory cytokines (Weisberg et al. 2003). For example, TNFα increases in adipose tissue promotes insulin resistance through serine phosphorylation of insulin receptor substrate 1 (Hotamisligil et al. 1996; Schenk et al. 2008). Also, these WAT-derived cytokines will modulate brain activity both directly by reaching the organ (Thaler et al. 2012) and through modulation of peripheral neuronal networks that send information on metabolic status to the CNS (Pirzgalska and Domingos 2018). Therefore, alterations in the metabolic information that reaches the CNS will in turn influence metabolic disease settlement and progression, associated with type 2 diabetes and non-alcoholic fatty liver disease, among others (Chait and den Hartigh 2020).

Moreover, adipocytes are the origin of chemokines and metabolically active mediators called adipokines. The adipokines, including adiponectin and leptin, have been demonstrated to signal through the hypothalamus to control whole-body metabolism, including modulation of sympathetic output to adipose tissue (Wang and He 2018) and directly modulate macrophage status in WAT, and thermogenesis in BAT during obesity (Cereijo et al. 2018; Larabee et al. 2020). Leptin, also known as the satiety hormone, has special importance in the context of immune system-neuronal control link in metabolic diseases, not only because hyperleptinemia is a hallmark of almost all metabolic diseases (Haynes et al. 1997) but also because both central and peripheral leptin levels and leptin receptor activity alterations are associated with sympathetic nervous system disarrangement and adipose tissue dysfunction (Ramseyer and Granneman 2016).

## Inflammation neuronal control: the hypothalamic-pituitary-adrenal axis and the vagal reflex

Two neuro-hormonal anti-inflammatory pathways have been described in more detail in the literature: the hypothalamic-pituitary-adrenal (HPA) axis and the vagal anti-inflammatory reflex (Fig. 1) (Pavlov et al. 2003). In reflexes involving the CNS, an environmental change activates the afferent (sensory) neurons that signal to CNS interneurons (integrative centers) and thus elicit a response via efferent neural outputs (Pavlov et al. 2018).

The HPA axis induces long-lasting anti-inflammatory responses to the whole body, but it is in general slow to respond to immediate inflammation. By contrast, the vagal anti-inflammatory reflex responds rapidly to inflammation and continuously ensures some attenuation of pro-inflammatory cytokine production (Chavan and Tracey 2017).

The HPA axis is activated by several stimuli including psychological stress, which activate the paraventricular nucleus (PVN) of the hypothalamus and leads eventually to the release of cortisol releasing hormone (CRH) into the anterior pituitary (Rivier and Plotsky 1986). In turn, CRH induces the release of adrenocorticotrophic hormone into the blood stream, which stimulates the production of glucocorticoids by the adrenal cortex (Fig. 1) (Keller-Wood and Dallman 1984). Glucocorticoids are potent anti-inflammatory molecules whose effect is mediated by the glucocorticoid receptor, a nuclear receptor expressed by almost all cells in the body and by innate immune cells in particular (Turnbull and Rivier 1999).

While the inhibition of pro-inflammatory cytokine production by immune cells is mediated by glucocorticoids when the HPA axis is activated, the vagal anti-inflammatory reflex relies on the binding of acetylcholine (ACh), the main neurotransmitter used by vagus nerve efferent fibers, and activation of nicotinic AChR (nAChR) (Fig. 1) (Borovikova et al. 2000).

The vagal anti-inflammatory reflex, also called the cholinergic anti-inflammatory reflex, is coordinated by peripheral vagal afferent nerves (Tracey 2002). The CNS activates vagal efferent nerves that synapse at the celiac ganglion to activate parasympathetic noradrenergic innervation of the spleen, leading to the release of NE (Andersson and Tracey 2012). The NE released binds to β2 adrenergic receptor (β2ARs) on the surface of T-cells positive to CD4, inducing the release of non-neuronal ACh, to thus reduce cytokine production and resolve inflammation through nAChR activation (Andersson and Tracey 2012). ACh released under vagal control interacts with α7nAChR on macrophages, an essential mediator of vagal anti-inflammatory reflex (Fig. 1) (Wang et al. 2003). Afferent vagus nerve arrives at the brainstem of the CNS and more specifically the NTS (Fernandez et al. 2014). The NTS, DMN (a major source of vagus nerve efferents) and the area postrema (proximal to the circumventricular organ) form the dorsal vagal complex (DVC), an important brainstem integrative and regulatory center (Berthoud and Neuhuber 2000).

Vagus activation is mediated by TNF, IL-1β, prostaglandins, serotonin, and other molecules released from immune cells. For example, injection of LPS, cytokines or other pathogens into mice and rats stimulates vagus nerve afferent signaling, which can be traced to the NTS and then to other brainstem and forebrain regions (Goehler et al. 1998; Marvel et al. 2004). Electrophysiological recordings of these cytokine-specific alterations in vagus nerve activity are not obtained in genetically modified mice in which the gene encoding TNF and IL-1β receptors has been deleted, or in mice in which the vagus nerve has been surgically transected (Steinberg et al. 2016).

Anti-inflammatory reflex responses through the vagus nerve have been described to be involved in several pathologies. For example, Pavlov and co-workers have described a dysfunction in ACh release mechanisms driven by increases in cytokine production during endotoxemia (Pavlov et al. 2006), that was reversed by electrical activation of the vagus nerve (Olofsson et al. 2015). Also, in IBD cases many groups have shown that GI inflammation is accelerated and aggravated by vagotomy in mice (Ghia et al. 2007). Moreover, colonic inflammation was reversed by vagus nerve stimulation, highlighting the therapeutic potential of vagus modulation (Meregnani et al. 2011).

The inflammatory vagus reflex, also called inflammatory axis, challenges generally held view that the sympathetic and parasympathetic nervous systems work in opposition, because in this instance the sympathetic and parasympathetic nervous system work in tight collaboration (Rosas-Ballina et al. 2011). For example, in myeloid cells, the anti-inflammatory pathway relies on adrenergic and nicotinic receptors acting in parallel (Guyot et al. 2019). By light sheet imaging of whole clarified spleen it has been shown that there is a dense network of catecholaminergic and cholinergic fibers across the spleen. Moreover, electrical stimulation of the splenic nerve was able to decrease cytokine secretion, an effect that was abolished by both propranolol and methyllycaconitine, but not by atropine showing that adrenergic receptors and α7nAChRs, but not muscarinic AChRs were involved in the immune control by these autonomic nerve fibers reaching the spleen (Guyot et al. 2019). The authors also found that this electrical stimulation of spleen nerves alleviated the clinical symptoms in a mouse model of RA (Guyot et al. 2019), highlighting the importance of this approach as a therapeutic modality for patients with IMIDs.

Unilateral cervical vagotomy has been crucial to the confirmation and discovery by Charles Serhan et al.. that the vagus nerve contributes to the resolution of inflammation (Mirakaj et al. 2014). Moreover, many studies on the inflammatory reflex and the use of electrical stimulation of the vagus nerve as a therapeutic for inflammatory diseases has led to a resurgence of interest in bioelectronic medicine (Birmingham et al. 2014). For example, a study reported significant clinical remission in five of seven patients with Crohn’s disease subjected to 6 months of treatment with an implanted device for vagus nerve stimulation (Bonaz et al. 2016).

As research continues, it is likely that additional circuits, systems and mechanisms for regulating inflammation will be revealed. In this regard, in the last decade evidence has emerged that points to the carotid body (CB) as a new player in the immunity-nervous system link and electrical modulation of the carotid sinus nerve (CSN) as a possible way to decrease systemic inflammation (Santos-Almeida et al. 2017).

## The carotid body: a new intervenient in the neuroinflammation control

Hermann and co-workers were the first to suggest that the systemic endotoxin response was not dependent on the vagus alone (Hermann et al. 2001). The authors described that LPS activation of DVC neurons was not affected by bilateral vagotomy and suggested that the NTS could be a primary central nervous system detector of cytokines, or another neural afferent pathway distinct from vagus nerves (Hermann et al. 2001).

Supporting the involvement of other afferent signals, we discuss in the next sub-section some studies showing the involvement of CB and CSN activity on the effects of inflammatory mediators, promoted by LPS, lysophosphatidic acid (LPA) or Ovalbumin (OVA).

### CB responses to inflammatory mediators

The CB is the largest paraganglion and the most vascularized organ in the body (Mascorro and Yates 1980), located in the region where the common carotid artery bifurcates to form the internal and external carotid arteries. This bilateral organ is a polymodal sensor, capable of detecting alterations in oxygen and carbon dioxide concentrations, as well as diverse mediators such as angiotensin II, leptin or insulin (Conde et al. 2014; Kumar and Prabhakar 2012). It comprises integrated units of chemoreceptor neural cells, glial cells, and vascular cells surrounded by connective tissue, which collectively constitute a highly adaptive chemosensory organ (Liu et al. 2009) innervated by the CSN (Verna 1997). Afferent CSN fiber inputs are integrated in the NTS, in a similar manner to vagal afferents (Fig. 1) (Finley and Katz 1992). The CB is a conglomeration of the type I (glomus) chemoreceptor cells, and type II (sustentacular) cells that are similar to glia. Type I cells are the most abundant cell type within the CB, with a minimum of 3 and up to a maximum of 8 for each type II cell in most of the species (De Kock and Dunn 1966; Verna 1979).

Infiltrations of macrophages into the CB have long been noticed, but the reason(s) for their presence has been overlooked for many years (Kumar and Prabhakar 2012). However, in recent years accumulating evidence suggests that the CB has an immunomodulatory function, in communicating peripheral immune status to the brain (Wang et al. 2002; Wang et al. 2006). It is also evident that the CBs exhibit histological features of acute inflammation, that in turn alter its function under conditions that mimic disease states such as sepsis and endotoxemia (Fernández et al. 2008). Reyes et al. found that bilateral carotid/sinus chemo-denervation after intraperitoneal administration of LPS suppressed both the LPS-induced increase in the number of c-Fos^+^ neurons within the NTS and the increased levels of plasma cortisol, suggesting that the CB performs a neuroimmunomodulatory function (Reyes et al. 2012).

Moreover, systemic inflammatory responses, like the one induced by pathogen-associated molecular pattern molecules (PAMPs), such as LPS endotoxin, increased tonic CB chemosensory activity and reduced its responsiveness to transient excitatory or depressant stimuli. These effects were also prevented by prior bilateral carotid neurotomy (Fernández et al. 2008). Furthermore, LPS-induced sepsis in rats submitted to carotid chemo/baro-denervation showed a blunted tachypneic response and enhanced tachycardia and hypotension as well as enhanced epinephrine and TNF-α responses, and earlier multiple organ dysfunction onset with a lower survival time compared with sham operated-LPS rats (Nardocci et al. 2015). Also, hyperoxia-induced reduction of CB chemosensory activity in animal models of sepsis was associated with higher plasma levels of pro-inflammatory cytokines and mortality in septic rats (Rodríguez-González et al. 2014). More recently, Santos-Almeida et al. showed that CSN electrical stimulation in conscious rats, modulates the innate immune response to LPS by attenuating the plasma levels of TNFα, IL-1β and IL-6 as well as by increasing the anti-inflammatory cytokine IL-10 (Santos-Almeida et al. 2017), and that this chemoreflex anti-inflammatory pathway was decreased by bilateral carotid chemoreceptor denervation and by the use of propranolol and methylatropine, blockers of sympathetic and parasympathetic pathways, respectively (Fig. 1) (Santos-Almeida et al. 2017). Additionally, LPA, an important mediator in allergen-induced asthmatic lung inflammation (Knowlden and Georas 2014) induced CB activation in a rat model of asthma (OVA-sensitized), an effect mediated by TRPV1 and LPA-specific receptors in the CB and that in turn activated parasympathetic (vagal) reflexes that led to acute bronchoconstriction (Jendzjowsky et al. 2018). All in all, these findings strongly support by hold the view that the CB supports an immune-CNS axis.

**Fig. 2:**
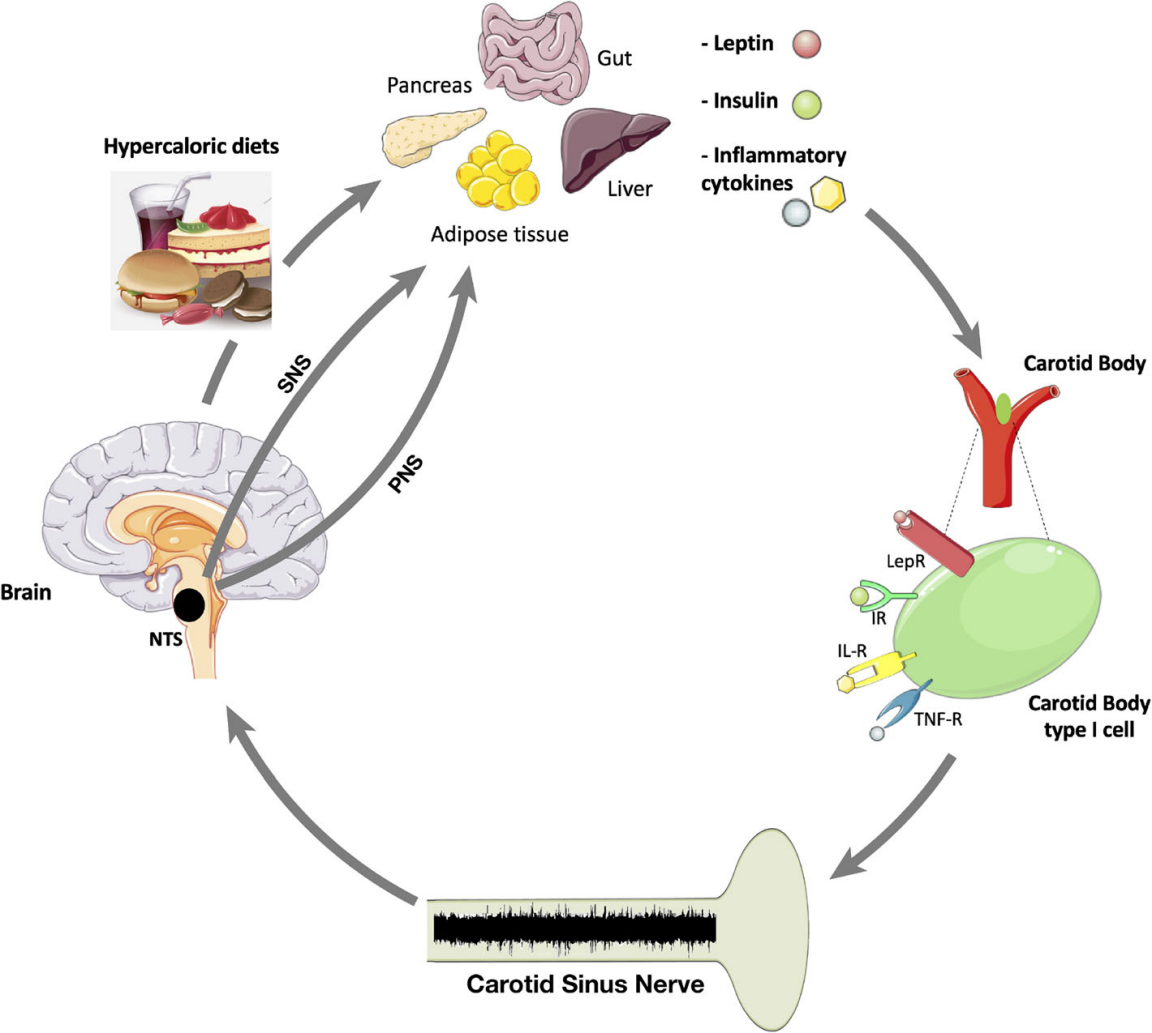
Postulated link between diet-induced dysmetabolism and the brain-carotid body axis: Hypercaloric diets generate a dysmetabolic state characterized by hyperleptinemia, hyperinsulinemia and inflammation, transduced by an overexpression of inflammatory cytokines. These key mediators contribute to carotid body (CB) dysfunction that is in the genesis of metabolic diseases since all these mediators are able to activate the CB, by acting on respective receptors within the organ, and modulate its function, e.g. via the release of neurotransmitters. Carotid sinus nerve (CSN) transmit this information from CB to the central nervous system, namely to the nucleus tractus solitarii (NTS), leading to the activation of both sympathetic and parasympathetic nervous systems to the efferent organs to modulate metabolism. Also, CSN information integrated in the NTS will impair satiety control and food intake, aggravating hyperphagia. All together, these afferent stimuli (metabolic vs inflammation) and intensity of reflexes stimulation may contribute to the activation or to different degrees of activation of different pathways contributing to dysmetabolic states

Therefore, a role in the response to some acute and chronic pathophysiological reactions to high levels of inflammatory and immune mediators in the bloodstream may originate from changes in CB function, its integration at the CNS level and/or in the control of sympathetic and parasympathetic efferents (Fig. 2).

### Presence and effects of inflammatory cytokines in CB and its outputs

TNF-α receptor expression in human, rat and mouse CBs was observed using microarray analysis (Mkrtchian et al. 2012) and western blot (Sacramento et al. 2020). Also, Fernandez et al. showed that the canonical LPS receptor TLR-4, and TNF-α receptors are functional (Fernández et al. 2008; Fernández et al. 2011). This cytokine and cytokine receptor expression on CB was not restricted to immune cells that infiltrated on this organ. In cats, in vitro recordings of the CSN activity showed that TNF-α administration did not modify basal CSN chemosensory activity but reduced in a dose-dependent manner the increase in the frequency of chemosensory discharge induced by hypoxia (Fernández et al. 2008). By contrast, in rats, TNF-α administration in dissociated type I cells increased [Ca^2+^]_i_ in response to acute hypoxia, an effect that was greater in cells obtained from the CB of rats exposed to chronic intermittent hypoxia (Lam et al. 2012) and chronic hypoxia (Lam et al. 2008). Additionally, TNF-α intravenous systemic administration in a dose of 5 ng/ml in the rat induced an increase in basal ventilation. This effect was abolished by CSN resection, demonstrating that TNF-α-induced increase in ventilation is mediated by the CB (Sacramento et al. 2020).

Glomus cells in the CB also displayed strong IL-1R (Wang et al. 2002) and IL-6Rα (Wang et al. 2006) immunoreactivity, and cultured rat CB type I cells responded to IL-6 administration. Therefore, there are specific receptor binding sites for cytokines on the CB type I cells, that support immune system-to-brain interactions (Fan et al. 2009). Also, studies of cultured type I cells harvested following 1 day of in vivo hypoxia showed elevated transcript levels of inflammatory cytokines and in situ hybridization studies confirmed expression of IL-6 in type I cells and also showed that chronic hypoxia induces IL-6 expression in supporting type II cells (Liu et al. 2009).

Additionally, IL-1β administration significantly increases CSN chemosensory discharge in anesthetized rats, an effect that was abolished by the administration of an IL-1β receptor antagonist (Shu et al. 2007). However, while IL-1β application did not modify the release of catecholamines from the rat CB, extracellular administration of IL-6 induced a rise in [Ca^2+^]_i_ and catecholamine release from in vitro-cultured CB type I cells (Fan et al. 2009). As described for TNF-α, the administration of IL-6 in the rat femoral vein in doses of 0.5 and 5 ng/ml augmented minute ventilation, an effect that was prevented by CSN denervation, suggesting that the effect of IL-6 on ventilation is CB-mediated (Sacramento et al. 2020).

Confirming that the CB participates in immune-brain signaling in humans, CBs from male volunteers exposed to hypoxia (10% O_2_) during a 1 h exhibit an increase in the IL-1β, IL-6, IL-8 and IL-10, whereas the release of IL-2, IL-5 and TNF-α could not be detected (Kåhlin et al. 2014). The same authors also showed that receptors for IL-1, IL-6 and IL-10 co-localize in human CB type 1 cells (Kåhlin et al. 2014).

Apart from relaying circulating immune signals to the brain in response to acute/intermediate systemic inflammatory conditions, the effect of proinflammatory cytokines on the CB has been proposed to be in the basis of CB overactivation that is associated with several diseases, such as obstructive sleep apnea and obesity that are associated with chronic systemic inflammation (Conde et al. 2017; Oyarce and Iturriaga 2018; Sacramento et al. 2020). Additionally, the role of CB on inflammation regulation blends with the autonomic reflex since CB stimulation provokes a wide array of cardiopulmonary and autonomic reflexes, as well as endocrine responses (e.g., plasma release of catecholamines and cortisol) (Fitzgerald 2014). Moreover, it is well described that diseases that are associated with high autonomic activity are associated with increased CB dysfunction (Paton et al. 2013), highlighting the link between inflammation – CB – autonomic reflex. For example, proinflammatory cytokines have been proposed as mediators of the chemosensory potentiation induced by chronic intermittent hypoxia (CIH), a condition that mimics obstructive sleep apnea (Del Rio et al. 2011; Iturriaga et al. 2009; Lam et al. 2012). Del Rio et al. found that ibuprofen, an anti-inflammatory drug, prevented the CIH-induced overexpression of cytokines in the CB and hypertension, but failed to block the enhanced CB chemosensory responses to acute hypoxia in Sprague-Dawley rats submitted to 21 days of intermittent hypoxia. These results show that proinflammatory cytokine levels may contribute to CIH-induced hypertension, highlighting another level of immunomodulation through the CB chemoreflex pathway (Del Rio et al. 2012). Another type of chronic hypoxia that also runs with chronic inflammation is chronic sustained hypoxia (Eltzschig and Carmeliet 2011). Chronic sustained hypoxia leads to profound morphological, biochemical and functional changes in the CB (Barnard et al. 1987; Bisgard 1994; Bisgard 1995; Bisgard 2000; Powell 2007; Powell et al. 2000) and is associated with the upregulation of the expression and function of proinflammatory cytokines in the rat CB (Lam et al. 2008). The expression of IL-1r1, gp130 and TNFr1 receptors was increased in the rat CB at 3, 7 and 28 days of chronic hypoxia, as were the gene transcripts of inflammatory mediators, inducible nitric oxide synthase and chemokines (MCP-1, CCR2, MIP-1alpha, and ICAM-1). Additionally, application of exogenous cytokines increased [Ca^2+^]_i_ responses to acute hypoxia in the dissociated fura-2-loaded glomus cells, a response that was significantly higher than in the normoxic group (Lam et al. 2008). Confirming that proinflammatory cytokines drive CB alterations that contribute to diseases associated with chronic systemic inflammation, common anti-inflammatory drugs (e.g. ibuprofen and dexamethasone) also reduced macrophage invasion, cytokine expression, and blocked the augmented CSN discharge induced by chronic hypoxia (Kumar and Prabhakar 2012; Liu et al. 2009). In addition to the above, pro-inflammatory cytokines and the transcripts of many anti-inflammatory cytokines (e.g. nuclear factor (NF)-κB, IL-10R and HMGB-1) have also been found in human and mouse CBs (Mkrtchian et al. 2012). It was also shown in human CBs that prolonged hypoxia releases not only pro-inflammatory cytokines but also anti-inflammatory cytokines, and that the corresponding cytokine receptors are expressed in the type I cells. In short, human CBs incorporate key components required to support immune system signaling (Kåhlin et al. 2014).

Apart from PAMPs, such as LPS, it was recently described that the CBs may also be activated by endogenous damage-associated molecular patterns, inflammatory mediators of aseptic tissue injury that are recognized by the same pathogen recognition receptors as PAMPs, including HMGB1 (all-thiol and disulfide forms) and S100 A8/A9 (MKrtchian et al. 2020). It was found that HMGB1, S100 A8/A9 and blood plasma from rats subjected to tibia surgery, a model of aseptic injury, induce the release of ATP and dopamine - key CB neurotransmitters – as well as TNF-α from ex vivo rat CBs (MKrtchian et al. 2020). Moreover, all-thiol HMGB1 and its reduced form, modulate several immune response genes including important pro-inflammatory cytokines, such as IL-1α and IL-1β (MKrtchian et al. 2020).

Recently our group have reviewed the key mediators that link CB dysregulation with the pathogenesis of metabolic diseases. We have demonstrated that these pro-inflammatory cytokines and also insulin and leptin are able to activate the CB and modulate its function. So, hyperinsulinemia, hyperleptinemia, and high pro-inflammatory cytokine levels seem to be determining factors that contribute to the CB overactivation in metabolic diseases (Fig. 2) (Sacramento et al. 2020).

Altogether these data support the view that CB function is regulated by endogenous mediators of innate immunity, and confirms once more that the CB is a key organ linking the immune system to the brain.

## The inflammatory reflex—linking immunity and metabolism

The vagus nerve, specially the efferent arm of the vagus nerve system, is a major constituent of the inflammatory reflex, or the efferent vagal anti-inflammatory reflex. In addition to its role in the regulation of metabolic homeostasis, the vagus is also crucial for the close relationship between the immune response and parasympathetic regulation of metabolism (Tracey 2002). This arm of immunity regulation is relayed by efferent nerves, that carry and array of brain outputs that modulate the innate immune system across the body (Fig. 1).

Inflammation is normally a temporary event that has to be resolved with achieving immune and physiological homeostasis. This anti-inflammatory reflex acts on the inflammation resolution by promoting anti-inflammatory molecules release like IL-10, TGF-beta and soluble cytokine receptors to stop proinflammatory progression (Pavlov and Tracey 2012a). Disruption of the vagal anti-inflammatory reflex action results in continual proinflammatory cytokines production and activity, and chronic inflammation, which contribute to the pathogenesis of inflammatory and metabolic diseases (Donath and Shoelson 2011; Hotamisligil 2017). It is well known that in situations of obesity, vagus nerve activity is decreased and interventions to activate cholinergic pathways in the vagus efferent arm of the inflammatory reflex have been shown to suppress obesity-associated inflammation and concomitant reversal of metabolic alterations (Carnethon et al. 2003; Marrero et al. 2010; Wang et al. 2011).

In the scenario of resolution of inflammation by the modulation of efferent vagus nerves within peripheral tissues, the α7nACh receptor is extremely important. The use of agonists of this receptor being identified as useful anti-inflammatory therapeutics (Pavlov et al. 2007; Wang et al. 2011). The work of Wang et al. (Wang et al. 2011) revealed that activation of this pathway by nicotine in obese animal models significantly improved insulin sensitivity and glucose homeostasis, and suppressed adipose tissue inflammation. Moreover, these beneficial effects were abolished in α7nAChR KO mice and pro-inflammatory cytokines expression was increased in this KO mice model (Wang et al. 2011).

The mechanoreceptors and chemoreceptors present in the afferent vagus nerves contribute to metabolic regulation by detecting changes in nutrients and metabolic molecules in the gastrointestinal tract, adipose tissue and the liver, such as glucose, lipids, insulin, leptin and other molecules, and relay information on their respective levels to the brain. Efferent vagus nerves, on the other hand, transmit the brain-derived outputs to the gastrointestinal tract, liver, pancreas and other tissues (Yi et al. 2010). For example, post-prandial state endotoxemia and inflammation have been proposed to be modulated by vagus nerve and inflammatory reflex. Intriguingly, TLR4 expression in the afferent vagus nerves after a meal provides a molecular sensory component that facilitates signalling to the brain in order to promote post-prandial inflammation resolution (de Lartigue et al. 2011; Pavlov and Tracey 2012b).

### Inflammatory reflex role on metabolism-related hormone activity

Cholecystokinin (CCK), is a hormone secreted by the enteroendocrine cells in the duodenum which stimulates the digestion of fat and proteins, which also acts on CCK1 receptors to inhibit gastric function, food intake and satiation in response to intestinal nutrients in humans (Little et al. 2005; Moran and Kinzig 2004). These effects are mainly mediated by the activation of the vagal afferents (Luyer et al. 2005) and probably modulated by the neuro-immune pathway. In fact, after a dietary lipid infusion, CCK acts both via vagus nerve afferents and directly in the brain to trigger efferent vagus nerve signaling, which in turn suppresses the release of proinflammatory cytokines and stops postprandial inflammation (Luyer et al. 2005). Ghrelin is another hormone secreted not only in the gastrointestinal tract but also in the pituitary, hypothalamus and pancreas among other organs, which plays a role in lipid metabolism, glucose homeostasis, growth hormone release, appetite stimulation, body weight gain, and adiposity whose mechanism of action seems to include its action on the anti-inflammatory vagal pathway (Ronveaux et al. 2015). The effects of ghrelin are mediated by central and peripheral mechanisms, its pre-eminent action being the activation of its receptors in the hypothalamus and activation of vagal afferents (Ronveaux et al. 2015). Electrophysiological studies reveal that, by contrast to CCK, ghrelin attenuates vagal activity. Therefore, these two hormones provide counterregulatory inputs to mechanisms that control metabolism, food intake and immunity (Date et al. 2002). Ghrelin, the release of which is stimulated by ACh, has known anti-inflammatory effects. Intravenous ghrelin administration in septic mice decreases the levels of the pro-inflammatory cytokines IL-1 and IL-6, and ghrelin contributes to the regulation of and its release is regulated by the vagus, in a manner partly coordinated by ghrelin receptors expressed in the DMN of the vagus, (Wu et al. 2007; Zhang et al. 2004).

Insulin and leptin are other hormones deeply involved in metabolic control (Date et al. 2002). Leptin acts on nodose ganglion of the vagus nerve increasing electrophysiological activity of vagal afferent nerves that contribute to energy homeostasis. Leptin resistance of vagal afferents leads to hyperphagia and an obesity phenotype (Ronveaux et al. 2015). By contrast to ghrelin action, leptin potentiates CCK function on food consumption and metabolism control (de Lartigue 2016). In obesity, the action of these appetite and metabolism-controlling hormones is impaired since LPS production in the gut is increased (DiBaise et al. 2008). LPS upregulates the expression of suppressor of cytokine signaling-3 that elicits leptin resistance through TLR4 expressed in the vagal afferent pathway (de Lartigue et al. 2011). Such leptin resistance occurs at the vagus nerve before it occurs at the hypothalamus in an obese animal model (de Lartigue et al. 2011). (Ueno and Nakazato 2016) have shown that mice fed with a high-fat diet develop inflammation in the intestinal tract as a result of changes in the intestinal flora, and that this inflammation spreads to the vagus nerve ganglion cells and the hypothalamus through the vagal afferent pathway distributed along the intestinal tract, leading to disrupted transmission of signals for appetite regulation (Naznin et al. 2015).

In addition to the above, insulin action and secretion is connected to changes in vagal neuron transmission to and receipt of information from the brain (Meyers et al. 2016). Nodose ganglion neurons of vagal afferents originating from the endocrine pancreas are activated by insulin and may inform about pancreatic insulin levels to the brain (Iwasaki et al. 2013). These fibers project to the NTS from where inputs are relayed to hypothalamic areas, such as the arcuate nucleus (ARC) and PVN, which are involved in the regulation of food intake and autonomic nervous system activity (Morton et al. 2006). Hepatic glucose metabolism is also regulated by vagal control of insulin action at this tissue, as revealed by studies on the effects of intracerebroventricular (ICV) insulin administration, the action of which was abolished by hepatic vagotomy (Pocai et al. 2005a; Pocai et al. 2005b). Hepatic inflammation is also regulated by central insulin action on efferent output through hepatic branches of vagal nerves, in part due to the fact that IL-6 is expressed in hepatic resident macrophages, Kupffer cells (Inoue et al. 2006).

Therefore, the activation of inflammatory pathways other than the vagus nerve interferes with insulin and leptin signaling in the brain and also in peripheral tissues, contributing to insulin resistance in obesity and metabolic disorders, such as type 2 diabetes.

### Inflammatory reflex in obesity

In obesity, chronic adipose tissue inflammation, sympathetic nerves and immune cells show an interconnection in the development of associated comorbidities. It is known that sympathetic nerves release NE in adipocytes promoting lipolysis and/or thermogenesis (Larabee et al. 2020). Advanced imaging techniques combined with optogenetics and pharmacologic approaches have allowed the visualization of the innervation of adipose tissue by the sympathetic nervous system and the confirmation that sympathetic innervation is necessary and sufficient to promote lipolysis in WAT (Zeng et al. 2015). Sympathetic innervation originates in five distinct brain regions, including the PVN, where leptin-mediated pro-opiomelanocortin (POMC) and agouti-related protein (AgRP) pathways converge. Adipose tissue homeostasis has also been described to be controlled by a neuroendocrine loop with leptin in the center of the orchestra acting on neural circuits in the hypothalamus and other regions in the brain to regulate food intake and peripheral metabolism (Friedman and Halaas 1998). Experimental leptin levels modify sympathetic output to adipocytes, leading to alterations in thermogenesis in BAT and lipolysis and/or browning in WAT (Shen et al. 2007). Central administration of leptin increases sympathetic activity on WAT promoting lipolysis, an effect prevented by the administration of the β-blocker propranolol, suggesting that leptin effects on WAT are mediated by NE binding to β-adrenergic receptors within adipose tissue (Shen et al. 2007). Therefore, circulating leptin levels have been shown to be proportional to adipose tissue size and function, and thus help maintain adiposity within a very narrow physiological range (Phillips et al. 2000). Despite our growing knowledge of the precise neural circuitry responsible for the control of sympathetic nervous system output, the specific mechanisms involved in the activation of leptin receptor-expressing neurons remains unclear, as is the path to peripheral and central leptin resistance (Haynes et al. 1997; Mahú and Domingos 2017).

Immune cells in adipose tissue also participate in the pathophysiology of obesity, with macrophages being key contributors in this context. Macrophage polarization is particularly relevant, as classically (M1-like) and alternatively (M2-like) activated macrophages are characteristic of obese and lean adipose tissue phenotypes (Wu et al. 2011). The hyperlipidemia characteristic of obesity inundates TLR4 in adipose tissue macrophages, which leads to an M1-like phenotype that includes release of pro-inflammatory cytokines, such as TNF and IL-6 (Osborn and Olefsky 2012; Shi et al. 2006). This stimulation of pro-inflammatory factors will aggravate insulin resistance and glucose intolerance linked with obesity due to the dysfunction of insulin signaling cascade activation and glucose transporter recruitment to the membrane (Osborn and Olefsky 2012). Recently, Pirzgalska et al. showed that adipose tissue macrophages are specific macrophages, called sympathetic neuron-associated macrophages (SAMs) that express neural- and adrenergic-related genes different from other macrophages and that produce NE following sympathetic activation (Pirzgalska et al. 2017). Also, they showed, in high fat diet obese mice, that these adipose tissue specific macrophages are augmented in obesity and promoted an increased clearance of NE (Pirzgalska et al. 2017), and that they are in higher number in visceral WAT than in subcutaneous adipose tissue or other fat pads (Mahú and Domingos 2017). Altogether these results suggest that these adipose tissue SAMs are the link between immune system and sympathetic activation in the modulation of adipose tissue in both physiologic and obesity conditions and confirm the visceral fat pad as being of huge importance for the maintenance of immune balance and metabolism.

Some studies have shown that gut and hepatic metabolism regulation are also modulated through the activation of vagal afferent pathways that innervate these tissues, and that they are also impaired in rodent models of type 2 diabetes (Lee et al. 2012). In fact, liver appears as a crucial target tissue for brain-immune axis control of metabolism. As already mentioned, central insulin action suppresses hepatic glucose production by downregulating the gene expression of gluconeogenic enzymes such as G6pc which encodes for glucose-6-phosphate (Kimura et al. 2016; Obici et al. 2002). In fact, the suppression of hepatic glucose production using the hyperinsulinemic-euglycemic clamp technique is abolished by insulin receptor deficiency, insulin receptor knockdown, and PI3-K inhibition in the hypothalamus (Inoue et al. 2006; Obici et al. 2002). Also, electrical stimulation of the vagus nerve has been shown to suppress the induction of inflammatory cytokine expression including IL-6 in the liver following LPS administration (Borovikova et al. 2000). This shows the role of hepatic vagal branches in the regulation of Kupffer cell action over the central insulin-mediated hepatic responses of IL-6/STAT3 signal and regulation of gluconeogenic enzyme gene expression (Kimura et al. 2016). Importantly, this regulatory mechanism is impaired in metabolic diseases such as obesity and type 2 diabetes (Taylor 1999). Additionally, catecholamine excess seems to be involved in hepatic glucose overproduction characteristic of obesity and other metabolic disorders, which suppress the induction of gluconeogenic enzymes (Nguyen et al. 2019). Moreover carvedilol, a third-generation beta-blocker and α1 adrenoceptor antagonist, blunted these hepatic dysfunctions in an animal model of high fat diet-induced obesity (Feuerstein and Ruffolo Jr 1995; Stoschitzky et al. 2001).

In conclusion, attenuation of vagus nerve signaling and consequently the anti-inflammatory reflex aggravates not only obesity and obesity-related metabolic disorders, but also the disarrangement of the sympathetic-immune link. Therefore, dysmetabolism and inflammatory pathway activation leads to a vicious cycle that accelerates many metabolic and inflammatory disorders (Pavlov and Tracey 2012a).

## The carotid body as a new player in the link between immunity and dysmetabolism

In recent years, CB overactivation has been associated with several cardiometabolic diseases, such as type 2 diabetes, essential hypertension and hypertension associated with obstructive sleep apnea (Conde et al. 2017; Fletcher et al. 1992; Narkiewicz et al. 1999; Ribeiro et al. 2013). These pathologies share an increase in sympathetic tone that is linked with CB and CSN hyperactivity, both of which contribute to pathogenesis (Conde et al. 2018; Dos Santos et al. 2018; Iturriaga 2018; Paton et al. 2013).

In 2013, Ribeiro et al. (Ribeiro et al. 2013) described for the first time that the CB is a peripheral regulator of insulin sensitivity and thus in the genesis of metabolic diseases, since bilateral CSN resection prevented the development of dysmetabolic changes induced by hypercaloric diets (Ribeiro et al. 2013). After that pioneering study, the same authors found that CSN resection normalized systemic sympathetic nervous system activity, insulin sensitivity and glucose tolerance and reversed weight gain induced by high-energy diets in prediabetes and early type 2 diabetes animals models (Sacramento et al. 2017), and in models of obesity (Melo et al. 2019) by improving glucose uptake by the liver and perienteric adipose tissue (Sacramento et al. 2017). In agreement with the hypothesis that CB overactivation promotes metabolic deregulation via an increase in sympathetic nervous system activity (Conde et al. 2017; Conde et al. 2014), the authors found that animal models of metabolic dysfunction induced by hypercaloric diets exhibit an overactivation of CB, by identifying associated increases in basal ventilation, ventilatory responses to ischemic hypoxia and CB chemoreceptor cell activity, as well as by the enlargement of the CB (Fig. 2) (Dos Santos et al. 2018; Ribeiro et al. 2013). CB dysfunction in metabolic disease states was consistent with the increased activity of the CSN electrophysiological recordings both in vivo (Cracchiolo et al. 2019b) and ex vivo (Conde et al. 2018) in the rat. Demonstrating that these mechanisms are also present in humans, Cramer et al. (Cramer et al. 2014) showed that patients with type 2 diabetes exhibit CBs 20–25% larger than control volunteers and more recently it was described that prediabetes patients exhibit increased CB activity (Cunha-Guimaraes et al. 2020), measured by the double-breath Dejours test, that evaluates CB chemosensitivity in patients while breathing 2 breaths of 100%O2 (Dejours 1962; Dejours 1963).

The important role of CB in both physiologic and pathological conditions is additionally supported by its metabolic sensing properties that have been highlighted in the last decades and that give the CB the capacity to respond to changes in circulating metabolic mediators, such as insulin and leptin (Fig. 2) (Prabhakhar and Joyner 2014). The emerging information on the role of CB as a metabolic sensor stands, naturally, on their anatomic location and crucial role as an alarm mechanism to the central nervous system in acute emergency situations that may lead to neuroglycopenia (Conde et al. 2018), as well as in chronic situations requiring maintenance of the balance between physiology and pathological resolution.

### Insulin role in CB activity

Insulin and leptin are potent sympathetic activators (Marino et al. 2011) whose activity in the brain have been highlighted as one of the major determinants of peripheral glucose and insulin responsiveness and lipid metabolism (Kimura et al. 2016). However, some old and recent evidences indicated that both these hormones might work outside the brain to activate autonomic efferent nerves. More than 50 years ago, Pereda et al. (Pereda et al. 1962) observed that insulin administration into the carotid artery of anesthetized dogs induced a higher increase in sympathetic activity than the systemic administration of insulin, suggesting a role for the peripheral nervous system in insulin-mediated increase in sympathetic nervous system activity. Some of the first evidences that insulin is capable of stimulating CB chemoreceptors came from studies dedicated to evaluate the CB glucose sensing properties and in where hypoglycemia was achieved through insulin administration (Bin-Jaliah et al. 2004; Ward et al. 2007). The effect of insulin per se on ventilation and on CB activity was later confirmed by Ribeiro et al. (Ribeiro et al. 2013) in anaesthetized rats, since insulin increased ventilation in a dose-dependent manner during an euglycemic clamp, an effect that it was absent in animals submitted to the CSN resection. Additionally, the authors demonstrated the existence of insulin receptors at the CB and its phosphorylation in response to insulin and that insulin, in physiological concentrations, is able to elicit a neurosecretory response from the CB, measured as the increase in [Ca^2+^]_i_ and the release of ATP and dopamine from the CB (Ribeiro et al. 2013). Insulin administered in vivo in the rat was also capable to increase CSN and sympathetic nervous system electrophysiological activities. This effect of insulin on sympathetic activity was prevented by CSN bilateral denervation (Cracchiolo et al. 2019a), meaning that the effect of insulin on the overall-sympathetic nervous system activity is mediated by the CB. The effects of insulin on ventilation were recently confirmed in humans. Minute ventilation increase during an hyperinsulinemic-euglycemic clamp and elevated plasma insulin levels increases minute ventilation independently of alterations in glucose levels (Barbosa et al. 2018), confirming the role of insulin as a contributor to CB dysfunction in metabolic diseases. Recently, it was described that CB increased activity is associated with increased circulating insulin levels and insulin resistance evaluated by HOMA-IR (Fig. 2) (Cunha-Guimaraes et al. 2020).

### Involvement of leptin signaling in CB activity

Leptin is another hormone that has been shown to activate the CB. Leptin may thus contribute to CB dysfunction and increased sympathetic activity seen is obesity and obesity-related syndromes, as OSA and metabolic syndrome (Fig. 2) (see e.g. (Kim and Polotsky 2020; Sacramento et al. 2020)). Leptin receptors are present in type I cells of mice, rat and human CBs (Caballero-Eraso et al. 2019; Messenger et al. 2012; Porzionato et al. 2011; Ribeiro et al. 2018). Within human CBs approximately 40% of type I cells were immunoreactive to leptin, with 57% of the type I cells immunoreactive to all leptin receptors isoforms, and approximately 30% immunoreactive for Ob-Rb isoforms (Porzionato et al. 2011). Acute exogenously applied leptin increased basal spontaneous ventilation as well as ventilatory responses to ischemic and hypoxic hypoxia in mice and rats, these effects of which were abolished or attenuated by CSN denervation (Caballero-Eraso et al. 2019; Olea et al. 2015; Ribeiro et al. 2018; Sacramento et al. 2020). In agreement with these excitatory effects of leptin on breathing mediated by the CB, exogenous leptin was also able to increase electrophysiological CSN activity measured ex vivo and in vivo (Caballero-Eraso et al. 2019; Ribeiro et al. 2018; Shirahata et al. 2015). CBs of insulin resistant rats submitted to a high-fat diet (60% energy from fat) for 3 weeks exhibited a higher degree of CB activation (increased expression of tyrosine hydroxylase, release of neurotransmitters and ventilation) with similar levels of hyperinsulinemia when compared with animals submitted to a high sucrose diet (Ribeiro et al. 2013). This led Conde et al. to hypothesize that a factor related to obesity could contribute to CB overactivation in metabolic diseases.

The effects of leptin on satiety (Dalamaga et al. 2013) are opposite to the acute and chronic effects of leptin mediated by the CB. Exogenous administration of leptin during 7 days did not modify basal ventilation but increased the hypoxic ventilatory response in rats (Yuan et al. 2018). In agreement with the lack of effects of leptin on baseline due to a higher tonic activity of the organ, rats submitted to a high-fat diet for 3 weeks exhibited increased basal ventilation (Ribeiro et al. 2018; Ribeiro et al. 2013) but showed decreased excitatory effects of leptin in spontaneous ventilation (Ribeiro et al. 2018; Sacramento et al. 2020), effects that were not modified with CSN resection (Sacramento et al. 2020). Also, leptin in these high fat rats was unable to increase electrophysiological sympathetic nervous system activity, measured at the cervical sympathetic chain, or the basal CSN electrophysiological activity (Ribeiro et al. 2018; Sacramento et al. 2020). Moreover, it was shown that in low-grade obese prediabetic animals the expression of leptin receptors was increased (Ribeiro et al. 2018) suggesting a feed-forward mechanism to promote CB activation during hyperleptinemic states. Altogether these results lead Conde et al. to postulate that leptin could be involved in the development of CB dysfunction in initial states of dysmetabolism that run with high leptin levels but before chronic hyperleptinemia is established (Fig. 2) (Ribeiro et al. 2018; Sacramento et al. 2020). In agreement with the development of a CB-leptin resistant state, rats exposed to hypercaloric diets during longer periods of time (8 weeks (Yuan et al. 2018), 16 weeks (Rakoczy et al. 2018) or 25 weeks (Sacramento et al. 2018)) show decreased spontaneous basal ventilation and decreased hypoxic ventilatory responses. These outcomes are similar to those observed with the obese Zucker rat, a model that lacks the gene coding for the Ob-R leptin receptor (Yuan et al. 2018).

Taking into account the close connection between insulin resistance, leptin resistance and dysmetabolism with chronic low-grade inflammation it becomes imperative to understand the mechanisms under the basis of this connection and the role of peripheral nervous system in this scenario. In fact, in an animal model of prediabetes, that combined insulin resistance, obesity and hypertension, obtained by submitting rats to a lipid-rich diet (60% energy from fat) for 3 weeks an increase in the IL-1 receptor in the CB was observed (Sacramento et al. 2020). Consistent with this increased inflammatory pattern, preliminary results showed that the expression of the receptor TNF-R1 in the CB increased in this animal model, although with no alterations on IL-6Rα receptor levels promoted by the high-fat diet. When the expression of the receptor for IL-1β, IL-6 and TNF-α in the CB was evaluated in a rat model of an early phase of type 2 diabetes, obtained by submitting the animals to a lipid rich diet combined with sucrose in the drinking water for 25 weeks, the expression of TNF-R1 receptor was increased, with no changes the expression of IL-6Rα and IL-1R. Altogether these results suggest that alterations in IL-1β and TNF-α signaling, and in the CB inflammatory sensing and reflex mechanisms might contribute to the increase in the CB activity and the altered link immunity-metabolism-nervous system observed in metabolic diseases (Fig. 2) (Cracchiolo et al. 2019a; Cracchiolo et al. 2019b; Cunha-Guimaraes et al. 2020; Ribeiro et al. 2018; Ribeiro et al. 2013). Knowing that the CB/CSN activate and interfere with both sympathetic (Fletcher et al. 1992; Marshall 1994; Sacramento et al. 2017; Zera et al. 2019) and parasympathetic nervous (Jendzjowsky et al. 2018) systems we can postulate that different stimuli (metabolic vs inflammatory) and intensity of stimulation may contribute to the activation or to different degrees of activation of different pathways contributing to dysmetabolic states (Fig. 2).

## Modulation of CSN and CB to control: implications in health and disease

Bioelectronic Medicine and electroceuticals-based medicines target the modulation of the electricity of the body to correct pathological conditions. This emerging and exciting field of research is supported by the fact that circuits made of neurons communicating through electrical impulses are the main regulators of all organs’ functions (Famm et al. 2013). Device-generated brain modulation, like deep brain electrical stimulation, transcranial magnetic stimulation, and transcranial direct current stimulation have been in clinical or exploratory use for various neurological conditions (Cabrera et al. 2014; Famm et al. 2013; Fregni and Pascual-Leone 2007). Vagus nerve stimulation (VNS) has also been used for the treatment of inflammatory diseases (Koopman et al. 2016), like IBD (Pavlov and Tracey 2015). For example, a study reported significant clinical remission, in five out of seven patients with Crohn’s disease, submitted to 6 months of treatment with VNS (Bonaz et al. 2016). Additionally, Koopman et al. developed the first clinical trial using VNS to treat RA, in which a set of 17 RA patients submitted to an 84 day open-label trial revealed significantly decreased TNF production and significantly improved clinical symptoms (Koopman et al. 2016).

### Modulation of vagus nerve activity for the resolution of metabolic diseases

The use of electrical stimulation of vagus nerve applied as a therapeutic for metabolic/inflammatory diseases envisioned and highlighted the importance of bioelectronic medicine not only for these pathologies but also for other therapeutic applications (Birmingham et al. 2014; Dirr et al. n.d.). Homeostatic and reward signaling of brain circuits has been implicated in obesity and metabolic disorders together with low firing capacity of vagal nerves (Atasoy et al. 2012; Fernandes et al. 2020; Sternson et al. 2013) and there is an increasing interest in the development of treatments aimed at targeting brain regions associated with appetite behaviors (Whiting et al. 2018). Neuromodulation using electrical or chemical alterations has been applied to improve biological functions and can either stimulate or inhibit central or peripheral autonomic nervous processes to influence homeostatic and reward pathways associated with metabolic diseases (Dendy et al. 2019). Regarding electrical modulation for obesity, deep brain stimulation has been tested in the hypothalamus, ventromedial hypothalamus, and nucleus accumbens with promising findings, although further research is needed to elucidate the long-term efficacy of this therapy (Whiting et al. 2018). VNS has also been tested for obesity and metabolic disorders, although there are discrepancies in the results reported by different studies. However, it is widely accepted that electrical stimulation of vagus nerve benefits individuals by increasing vagus nerve signaling pathways and increasing satiety (Pelot and Grill 2018). In fact, vagus nerve modulation (vBloc therapy) is currently an FDA approved treatment for long-term treatment of obesity in patients aged from 18 to 65 years with a body mass index (BMI) of 40 to 45 kg/m2 or > 35 kg/m2 and one or more obesity-related complications. Results from the Recharge study described that after 12 months patients with vBlock activated lost 8.5% more weight than the controls (Morton et al. 2016). Also, although safe, the vblock therapy had several adverse effects such as nausea, pain at the neuroregulator site, vomiting, and surgical complications as well as heartburn, problems swallowing, belching, mild nausea, and chest pain (Morton et al. 2016). In vBloc therapy both afferent and efferent vagal nerves are blocked and it has been postulated that its effects are due to changes in satiety and consequent weight loss (Dendy et al. 2019; Ikramuddin et al. 2014).

The mechanism behind vagus nerve stimulation for the treatment of obesity and metabolic complications has been associated with regulation of peristaltic movements and distension and compression of gut and stomach, respectively, with the regulation of central firing in regions dedicated to satiety and food intake control, and normalization of leptin resistance (de Lartigue 2016). However, since caloric intake has been described as unaltered in many studies that show body weight decrease and metabolic homeostasis in other peripheral organs, it is crucial that we understand other mechanisms that might confer the long-term beneficial effects of vagal electrical treatment in weight loss, glucose homeostasis and metabolic control (Pelot and Grill 2018). On this scenario we postulate that the previously described role of vagal reflex on immune regulation both in peripheral organs and brain can be responsible for some beneficial effects of this therapeutic technique. This hypothesis has been tested with some important and promising results like the work of Zang et al. that showed that vagus electrical stimulation decreased overall inflammation and autonomic control of weight (Zhang et al. 2009).

### CB and CSN modulation for the treatment of metabolic diseases

As a therapeutic target, the electrical modulation of CB chemoreceptors could also modify the inflammatory response during sepsis syndromes through a network consisting of neural - sympathetic and parasympathetic activation and, as a consequence, cytokine elements. Santos-Almeida et al. clearly stated that, in conscious rats, CSN electrical stimulation (1 mA, 1 ms, 30 Hz) during 1 h, attenuates the response to LPS by decreasing TNF, IL-1β and IL-6 levels and increasing the levels of IL-10, effects mediated by both sympathetic and parasympathetic pathways (Santos-Almeida et al. 2017). CB electrical modulation have also been tested for metabolic diseases, with Sacramento et al. showing that kilohertz frequency alternating current (KHFAC) modulation, and therefore electrical blocking applied continuously to the CSN was able to restore after 1 week, and maintain during 9 weeks insulin sensitivity and glucose tolerance in an early type 2 diabetes animal model (Sacramento et al. 2018). Moreover, the authors found that KHFAC effects were reversible, as upon cessation of electrical modulation insulin resistance and glucose intolerance returned to normal values within 5 weeks (Sacramento et al. 2018). Driving KHFAC of the CSN towards clinical translation, Fjordbakk et al. showed in the pig that high frequency electrical modulation of the CSN was able to block evoked chemo-afferent responses (Fjordbakk et al. 2019). The CSN conveys not only the information that comes from the CB but also the baroreceptor information that comes from the carotid sinus, that is involved in the acute adjustments of blood pressure (Marcus et al. 2014). In fact, electrical stimulation of carotid sinus baroreflex afferents acutely decreased arterial blood pressure in hypertensive patients, an effect mediated through sympathetic inhibition (Heusser et al. 2010), with the Barostim Therapy® being approved by the FDA to treat drug-resistant hypertension and heart failure (FDA 2020). More recently, it was shown that electrical carotid baroreceptor stimulation by electrodes placed into the carotid sinus wall (10 Hz, 1 ms, stimulating cycle with an interval of 1 min each 5 min) improves glucose metabolism in obese rats by ameliorating adipose tissue function, particularly the BAT (Cao et al. 2019). In contrast with these results, May et al. (May et al. 2014) reported that in hypertensive patients baroreceptor stimulation did not elicit significant changes in muscle glucose delivery and whole-body insulin sensitivity, suggesting that baroreflex stimulation does not affect glucose metabolism and insulin action. Also, Wallbach et al. (Wallbach et al. 2015) showed that in 30 patients with drug-resistant hypertension (10 of those with diabetes) that long-term electrical stimulation of baroreflex during 6 months did not change significantly post-prandial glucose tolerance, fasting insulin levels, C-peptide levels, hemoglobin A1c, HOMA-IR, HOMA-β, ISQuickI, weight, and BMI. The differences between the findings of Cao et al. (Cao et al. 2019) work, May et al. and Wallbach et al. may rely on the different species studied – human vs rat – but we can postulate that probably in the studies of Cao et al. an interference with the CB chemoreceptors might have occurred. In fact, one could speculate that electrical current delivered to the baroreceptors by Cao et al. could have affected the CB chemoreceptors, therefore eliciting an anti-inflammatory response from the CB that would attenuate dysmetabolism. In agreement with a possible influence of CB chemoreceptors in BAT metabolism and with the interference of CB chemoreceptors in the effects of electrical modulation of baroreceptors observed by Cao et al. (Cao et al. 2019), it was previously shown that activation of CB chemoreceptors inhibits the elevated levels of BAT sympathetic nerve activity evoked by hypothermia (Madden and Morrison 2005). To discard the interference between the CB chemoreceptors and baroreceptors Cao et al. (Cao et al. 2019) should have evaluated basal ventilation, hypoxic ventilatory response and the baroreflex. Although, we cannot exclude a possible direct role of baroreceptors in attenuating inflammation and dysmetabolim or even a crosstalk between carotid chemo and baroreceptors in the modulation of this immuno-metabolic link, we can certainly suggest that carotid sinus nerve electrical modulation can be an approach for the treatment of metabolic diseases.

## Conclusion and perspectives

In recent decades several lines of evidence have highlighted the possibility of using the CB as a new point of intervention for neuroinflammation control. Supporting this, the CB has been described to be able to sense several cytokines (Fernandez et al. 2014; Fernández et al. 2011; Sacramento et al. 2020) being involved in the response to PAMPs, as LPS (Fernández et al. 2008; Kåhlin et al. 2014; Mkrtchian et al. 2012; Nardocci et al. 2015) and to endogenous damage-associated molecular patterns (MKrtchian et al. 2020). Additionally, our group has clearly demonstrated that the CB is a regulator of peripheral insulin sensitivity and glucose homeostasis and that CB dysfunction is involved in the genesis of metabolic diseases, and postulated that the modulation of CB/CSN activity might be used for the treatment of metabolic diseases as obesity and type 2 diabetes (Cunha-Guimaraes et al. 2020; Ribeiro et al. 2018; Ribeiro et al. 2013; Sacramento et al. 2018; Sacramento et al. 2017). CB dysfunction in metabolic diseases is associated with increased circulating levels of insulin (Conde et al. 2018; Cunha-Guimaraes et al. 2020; Ribeiro et al. 2013), leptin (Caballero-Eraso et al. 2019; Ribeiro et al. 2018) and inflammatory mediators such as cytokines released from adipose tissue (Conde et al. 2017). These mediators acting on the CB, via specialized receptors, modulate its function by, for example, altering the release of neurotransmitters from CB type 1 cells, and therefore changing the activity of CSN, the CB sensitive nerve (Fernandez et al. 2014; Fernández et al. 2011; Sacramento et al. 2020). The CSN integrates this information from the CB and relays afferent inputs to the NTS, leading to the activation of both sympathetic and parasympathetic nervous systems that modulate metabolism through efferent outputs to target organs, thus generating a vicious cycle between dysmetabolism and inflammatory pathways that we postulate to be a contributing factor in metabolic diseases (Fig. 2).

Many of the studies that disclosed the role of CB as a sensor of inflammation and metabolism, its role in the genesis of metabolic diseases and the existence of an immune-metabolic CB-brain axis relied upon surgical ablation/resection of the CB/CSN as an experimental approach. Unilateral CB surgical resection has been tested in the past in patients with asthma (Anderson et al. 1986) and more recently resistant hypertension (Narkiewicz et al. 2016), although with disappointing results due to the lack of long-term efficacy due to CSN nerve fiber regeneration and the appearance of side effects (Conde 2018). Adding to this there are issues with patient compliance due the extreme nature of this invasive procedure. Therefore, a suitable and selective therapeutic strategy to modulate CB function is still lacking. Our group has however shown that continuous KHFAC very effectively mimics the positive outcomes of CSN resection in terms of glucose homeostasis and insulin action in the rat (Sacramento et al. 2018). This is an open door for further investigation of the use of bioelectronic medicine for the treatment of metabolic diseases. Feasibility of KHFAC modulation of CSN activity has been confirmed in the pig (Fjordbakk et al. 2019) showing its translational potential. An advance for this kind of therapeutic will be the development of closed-loop neuromodulation solutions to adjust in real-time the neural activity related to dysmetabolism and re-establish, as much as possible, the physiological conditions. Interestingly, Cracchiolo et al. recently showed that CSN activity strongly correlates with perturbations in glycaemia levels in both control and diabetic rats, indicating that CSN activity could serve as a marker to monitor glycaemic alterations and, therefore, could be used for closed-loop control of CSN neuromodulation (Cracchiolo et al. 2019a). Such progress is of course an important contribution to effective bioelectronic therapies. However, a more detailed neuronal decode of CSN activity in different disease states, and the characterization of the neural-immune-metabolic circuities involving the CB in the different metabolic diseases, is needed to allow for selective, adaptable and personalized bioelectronics therapies.

## Data Availability

Data sharing is not applicable to this article as no datasets were generated or analyzed during the current study.

## Abbreviations

ACh: Acetylcholine
AChRs: Acetylcholine receptors
AgRP: Agouti-related protein
AP: Area prostema
ARC: Arcuate nucleus
ARs: Adrenergic receptors
BAT: Brown adipose tissue
BBB: Blood-brain barrier
CB: Carotid body
CCK: Cholecystokinin
CIH: Chronic intermittent hypoxia
CNS: Central nervous system
CRH: Cortisol releasing hormone
CSN: Carotid sinus nerve
CVOs: Circumventricular organs
DCs: Dendritic cells
DMN: Dorsal motor nucleus of the vagus
DVC: Dorsal vagal complex
FDA: Food and drug administration
HPA: Hypothalamic-pituitary-adrenal
IBD: Inflammatory bowel disease
ICV: Intracerebroventricular
IFN-γ: Interferon-gamma
IL-1β: Interleukin-1beta
IL-6: Interleukin 6
IL-10: Interleukin 10
IMIDs: Immune-Mediated Inflammatory Diseases
KHFAC: Kilohertz frequency alternating current
LPA: lysophosphatidic acid
LPS: Lipopolysaccharide
nAChR: nicotinic Ach receptors
NE: Norepinephrine
NK: Natural killer cells
NTS: Nucleus tractus solitarii
PAMPs: Pathogen-associated molecular pattern molecules
POMC: Pro-opiomelanocortin
PVN: Paraventricular nucleus
RA: Rheumatoid arthritis
SAMs: Sympathetic neuron-associated macrophages
SLE: Systemic Lupus Erythematosus
vBloc therapy: vagus nerve modulation
TLRs: Toll-like receptors
TNF: Tumor necrosis factor
UCP-1: Uncoupling protein-1
VNS: Vagus nerve stimulation
WAT: White adipose tissue
